# The triglyceride glucose index is a simple and low-cost marker associated with atherosclerotic cardiovascular disease: a population-based study

**DOI:** 10.1186/s12916-020-01824-2

**Published:** 2020-11-25

**Authors:** Sangmo Hong, Kyungdo Han, Cheol-Young Park

**Affiliations:** 1 Department of Internal Medicine, Guri Hospital, College of Medicine, Hanyang University, Seoul, Republic of Korea; 2 Department of Statistics and Actuarial Science, Soongsil University, Seoul, Republic of Korea; 3 Department of Internal Medicine, Kangbuk Samsung Hospital, Sungkyunkwan University School of Medicine, Pyung-Dong, Jongro-Gu, Seoul, 03181 Republic of Korea

**Keywords:** Cardiovascular disease, Insulin resistance, Myocardial infarction, Stroke, TyG index

## Abstract

**Background:**

The triglyceride glucose (TyG) index is an inexpensive clinical surrogate marker for insulin resistance. However, the relationship between TyG index and atherosclerotic cardiovascular disease (CVD) remains unclear. We evaluated the relationship between TyG index and CVD using a large-scale population dataset from the National Health Information Database (NHID).

**Methods:**

We performed a retrospective observational cohort study of 5,593,134 persons older than 40 years from 2009 to 2017 using the NHID. We divided the participants into TyG index quartiles. Outcome variables were stroke, myocardial infarction, and both. The incidence of outcomes was estimated for each TyG quartile over the total follow-up period. All outcomes were analyzed by Cox proportional hazards regression analysis while controlling for baseline covariates.

**Results:**

During 8.2 years of mean follow-up, stroke was diagnosed in 89,120 (1.59%), MI in 62,577 (1.12%), and both stroke and MI in 146,744 (2.62%) participants. Multivariate-adjusted hazard ratios (HRs) for patients in the highest TyG index quartile demonstrated that these patients were at higher risk for stroke (HR = 1.259; 95% confidence interval [CI] 1.233–1.286), for MI (HR = 1.313; 95% CI 1.28–1.346), and for both (HR = 1.282; 95% CI 1.261–1.303) compared with participants in the lowest TyG index quartile. These effects were independent of age, sex, smoking, alcohol consumption, physical activity, body mass index, systolic blood pressure, and total cholesterol.

**Conclusions:**

In our large population study, TyG index, a simple measure reflecting insulin resistance, was potentially useful in the early identification of individuals at high risk of experiencing a cardiovascular event.

**Supplementary information:**

The online version contains supplementary material available at 10.1186/s12916-020-01824-2.

## Background

Atherosclerotic cardiovascular disease (ASCVD) is the second leading cause of death in Korea, suggesting the importance of prevention and early prediction of ASCVD [1]. In the prevention of ASCVD, various conditions, including diabetes, chronic kidney disease, peripheral artery disease, and stroke, have been considered indicative of high risk. Most guidelines encourage an individual-based approach focused on traditional cardiovascular risk factors implemented in risk stratification models [2–4]. In clinical practice, we have frequently encountered patients with new ASCVD events who had been misclassified by models based on traditional cardiovascular risk factors. For that reason, there is a need for better CVD risk prediction.

Insulin resistance represents a major underlying abnormality driving ASCVD [5]. Insulin resistance causes arteriosclerosis in part due to the associated chronic hyperinsulinemia. Chronic hyperinsulinemia increases very low-density lipoprotein (LDL) cholesterol synthesis, vascular smooth muscle cell growth and proliferation, and LDL cholesterol transport into arterial smooth muscle cells, as well as activating the genes involved in inflammation [6]. However, current risk stratification models do not include measures of insulin resistance that might improve the prediction of ASCVD. The triglyceride glucose (TyG) index, which is calculated using fasting triglyceride (TG) and fasting blood glucose (FBG) measurements, has been proposed as a simple and low-cost marker of insulin resistance [7]. Several studies showed that the TyG index is associated with ASCVD risk factors including type 2 diabetes [8, 9], hypertension [10], and metabolic syndrome [11]. The TyG index is also is associated with other surrogate markers of CVD including coronary artery calcium score [12], intima media thickness, and arterial stiffness [13, 14]. A few studies demonstrated that the TyG index is associated with CVD in high-risk patients such as those with diabetes and chronic kidney disease [15, 16]. However, few studies have evaluated the relationships among TyG index, insulin resistance, and incident CVD [17, 18].

In the current study, we evaluated potential relationships between TyG index and cardiovascular disease events using a large-scale population dataset from the National Health Information Database (NHID).

## Methods

### Study database

Data for our analysis were from the National Health Information Database (NHID), a public database on healthcare utilization and health screening that contains sociodemographic and mortality information for the entire population of South Korea. The NHID, which is produced by the National Health Insurance Service (NHIS), was launched in 2000 by integrating 375 insurance associations and contains data for the years 2002 to 2017. It provides longitudinal data for 97% of the Korean population with linkage to the National Death Registry and the national health screening program [19, 20]. This latter program was initiated in 2009 and includes a medical interview and postural examination, chest X-ray examination, blood test (including fasting glucose and triglyceride level), urine test, dental screening, and additional functions. Approval for the study protocol was obtained from the Institutional Review Board of Gangbuk Samsung Hospital (KBSMC 2018-01-036). And informed consent was waived by the board.

### Study participants

This was a national observational cohort study that included 5,593,134 persons. In total, 7,183,262 persons aged 40 years and older participated in the national health screening program in 2009 and were in the NHID database. Of these, 1,315,443 individuals who took anti-diabetic or lipid-lowering medications, 186,551 individuals lacking complete data, and 88,134 patients with a history of ASCVD were excluded from our study. Therefore, the total number of eligible participants was 5,593,134.

### Definitions of TyG index and study outcomes (cardiovascular events)

The TyG index was calculated as ln [TG (mg/dL) × FBG (mg/dL)/2], derived from previous studies [21, 22]. The outcomes of the study were newly diagnosed myocardial infarction (MI), stroke, or both. Stroke was defined as ICD-10 code I63 or I64 during hospitalization with claims for brain magnetic resonance imaging or brain computerized tomography, and MI was defined as ICD-10 code I21 or I22 during hospitalization. Participants were considered to have completed the study at the date of onset of cardiovascular event, or until December 31, 2018, whichever came first.

### Clinical and laboratory measurements

All participants completed a questionnaire on medical history, use of tobacco and alcohol, and exercise habits. Smoking habits were categorized as non-smoker, ex-smoker, or current smoker. Alcohol habits were classified as non-drinker, moderate drinker (< 30 g per day), or heavy drinker (≥ 30 g per day). Regular exercise was defined as vigorous-intensity exercise three or more times per week or moderate-intensity exercise five or more times per week. Low socioeconomic status was defined as income in the lowest 20% of the population. BMI was calculated as body weight (in kilograms) divided by height (in meters squared). Blood pressure (BP) was measured through the standard procedure with a sphygmomanometer after resting for more than 5 min. Blood samples were collected after overnight fasting. Serum glucose, total cholesterol, triglycerides (TG), high-density lipoprotein (HDL) cholesterol, and low-density lipoprotein (LDL) cholesterol were measured. We calculated glomerular filtration rate using the four-variable Modification of Diet in Renal Disease Study equation [23]. Baseline comorbidities included hypertension (ICD-10 codes I10 to I13 or I15 and treatment with antihypertensive medications, systolic BP ≥ 140 mmHg, or diastolic BP ≥ 90 mmHg), type 2 diabetes (ICD-10 codes E11 to E14 and antidiabetic drugs or fasting glucose level ≥ 126 mg/dL), hyperlipidemia (ICD-10 code E78 with lipid-lowering agents or serum total cholesterol ≥ 240 mg/dL), and chronic kidney disease (CKD; estimated glomerular filtration rate < 60 mL/min/1.73 m^2^).

### Data analyses

Baseline characteristics were analyzed using descriptive statistics. Categorical variables were described as frequency and percentage. Continuous variables were described as mean (± standard deviation [SD]) for normally distributed data and as geometric mean and 95% confidential interval (CI) for data not normally distributed. We compared the baseline TyG index quartile characteristics of participants. Continuous variables were compared using one-way ANOVA, while categorical variables were compared using the chi-square test. The follow-up duration of each TyG quartile group was obtained. The incidence rates of stroke, MI, and both were estimated for each TyG quartile over the total follow-up period. Incidence curves were estimated using the Kaplan–Meier method, and the log rank test was also conducted. All outcomes were analyzed by Cox proportional hazards regression analysis while controlling for baseline covariates. We deemed two-tailed *p* value less than 0.05 to be significant. Analyses were performed with SAS 9.4 (SAS Institute, Cary, NC, USA) and R program, version 3.4.1 (The R Foundation for Statistical Computing, Vienna, Austria, http://www.R-project.org).

## Results

### Baseline characteristics of study participants by TyG index

The baseline clinical and biochemical characteristics of the participants by TyG index quartile are shown in Table 1. Among all participants, TyG index quartile was positively associated with ASCVD risk factors and components of metabolic syndrome comprising age, BMI, waist circumference, current smoking, alcohol consumption, systolic and diastolic blood pressure, fasting glucose, total cholesterol, LDL cholesterol, triglycerides, and high prevalence of T2D, hypertension, dyslipidemia, and CKD (all *p* < 0.001). Regular physical activity, low socioeconomic status, and HDL cholesterol were negatively associated with TyG index quartile (all *p* < 0.001).

**Table 1:** Characteristics according to quartile of TyG index

	TyG index quartiles				*p* value
	Q1	Q2	Q3	Q4	
*N*	1,398,211	1,398,062	1,398,102	1,398,759	
Age (yeas)	51.62 ± 9.83	52.74 ± 10.04	53.59 ± 10.17	54.12 ± 10.26	< 0.001^†^
≧ 65	176,028 (12.59)	204,473 (14.63)	228,776 (16.36)	245,491 (17.55)	< 0.001^†^
Sex (male; *n*)	706,479 (50.53)	706,813 (50.56)	706,287 (50.52)	706,769 (50.53)	0.9243
Body mass index (kg/m^2^)	22.64 ± 2.69	23.42 ± 2.83	24.1 ± 2.9	24.89 ± 2.95	< 0.001^†^
≧ 25 kg/m^2^	250,943 (17.95)	375,229 (26.84)	494,765 (35.39)	645,970 (46.18)	< 0.001^†^
Waist circumference (cm)	77.19 ± 7.97	79.52 ± 8.22	81.47 ± 8.2	83.69 ± 8.01	< 0.001^†^
Men ≧ 90 and women ≧ 85	130,078 (9.3)	216,825 (15.51)	307,971 (22.03)	434,265 (31.05)	< 0.001^†^
Regular physical activity	310,405 (22.2)	284,886 (20.38)	269,826 (19.3)	249,608 (17.84)	< 0.001^†^
Low socioeconomic status	387,421 (27.71)	378,852 (27.1)	372,515 (26.64)	375,476 (26.84)	< 0.001^‡^
Smoking					< 0.001^†^
Non-smoker	926,718 (66.28)	894,479 (63.98)	871,643 (62.34)	838,042 (59.91)	
Ex-smoker	216,828 (15.51)	215,518 (15.42)	214,958 (15.37)	204,220 (14.6)	
Current smoker	254,665 (18.21)	288,065 (20.6)	311,501 (22.28)	356,497 (25.49)	
Alcohol drinking					< 0.001^†^
None	799,786 (57.91)	797,857 (57.74)	790,344 (57.2)	758,877 (54.89)	
Moderate	505,002 (36.57)	495,360 (35.85)	487,941 (35.31)	482,949 (34.93)	
Heavy	76,276 (5.52)	88,572 (6.41)	103,528 (7.49)	140,726 (10.18)	
Systolic blood pressure (mmHg)	119.46 ± 14.63	122.09 ± 14.85	124.3 ± 15	127.27 ± 15.33	< 0.001^†^
Diastolic blood pressure (mmHg)	74.45 ± 9.89	76.14 ± 9.95	77.52 ± 9.98	79.37 ± 10.13	< 0.001^†^
Glucose (mg/dL)	89.09 ± 10.78	93.2 ± 11.89	96.48 ± 13.79	105.09 ± 25.23	< 0.001^†^
Total cholesterol (mg/dL)	184.69 ± 30.82	194.79 ± 31.85	202.22 ± 33.17	211.45 ± 36.25	< 0.001^†^
HDL cholesterol (mg/dL)	60.22 ± 16.69	56.83 ± 17.29	53.98 ± 18.81	50.13 ± 22.57	< 0.001^†^
LDL cholesterol (mg/dL)	111.91 ± 28.82	118.8 ± 30.36	121.66 ± 31.92	115.37 ± 36.46	< 0.001^†^
Triglycerides* (mg/dL)	60.77 (60.74–60.79)	94.78 (94.75–94.81)	132.28 (132.24–132.33)	222.65 (222.51–222.79)	< 0.001^†^
Type 2 diabetes	6080 (0.43)	17,499 (1.25)	39,487 (2.82)	145,799 (10.42)	< 0.001^†^
Hypertension	253,013 (18.1)	332,933 (23.81)	407,314 (29.13)	511,203 (36.55)	< 0.001^†^
Dyslipidemia	60,164 (4.3)	112,443 (8.04)	173,081 (12.38)	279,795 (20)	< 0.001^†^
Chronic kidney disease	63,434 (4.54)	78,204 (5.59)	91,013 (6.51)	109,586 (7.83)	< 0.001^†^

*Geometric mean

^†^All *p* < 0.001 between TyG index quartiles by analysis of variance post hoc analysis with the Bonferroni method

^‡^Q3 vs. Q4; *p* = 0.001 and rest of the comparisons between quartiles all *p* < 0.001 between TyG index quartiles by analysis of variance post hoc analysis with the Bonferroni method

### Risk of incident stroke, myocardial infarction, and both according to TyG index quartile

**Fig. 1:**
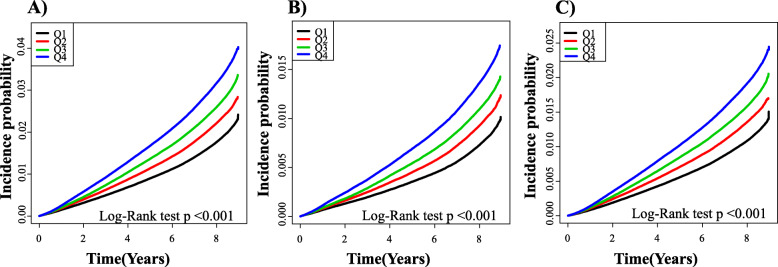
The cumulative incidence of stroke, myocardial infarction, and both by Kaplan-Meier analysis. Occurrence of **a** myocardial infarction and stroke, **b** myocardial infarction, and **c** stroke during follow-up grouped according to TyG index quartile. Cumulative incidence probability is presented on the *y*-axis. Plots use different *y*-axis scales. The *p* value was calculated with the log-rank test

Among the 5,593,134 participants, 2,826,348 (50.5%) were men and 2,766,786 (49.5%) were women. During 45,678,299 person-years of follow-up, there were 146,744 incident cases of MI and stroke (overall incidence of 2.62% or 3.21 cases/1000 person-years). There were 89,120 incident cases of stroke (overall incidence of 1.59% or 1.94 cases/1000 person-years) during 45,878,021 person-years of follow-up and 62,577 incident cases of MI (overall incidence of 1.12% or 1.36 cases/1000 person-years) during 45,987,003 person-years of follow-up. Figure 1 shows the Kaplan-Meier curves for cumulative incidences of stroke, MI, and both for TyG index quartiles. The highest TyG index quartile was associated with the highest probability of developing incident stroke, MI, and both; these probabilities decreased sequentially for lower quartiles (all log rank *p* < 0.001, Fig. 1). The age- and sex-adjusted hazard ratio (HR) for MI and stroke was increased for the 2nd (1.180, 95% CI 1.161–1.199), 3rd (1.338, 95% CI 1.317–1.359), and 4th (1.608, 95% CI 1.584–1.633) TyG index quartiles compared to the 1st quartile (*p* for trend < 0.001, Table 2). Age- and sex-adjusted HRs for MI increased across TyG index quartiles: 1.204 (95% CI 1.174–1.234), 1.379 (95% CI 1.346–1.412), and 1.697 (95% CI 1.658–1.737) for the 2nd, 3rd, and 4th quartile, respectively, compared with the 1st quartile (*p* for trend < 0.001, Table 2). Stroke also increased with increasing TyG index: 1.164 (95% CI 1.14–1.188), 1.306 (95% CI 1.28–1.333), and 1.545 (95% CI 1.515–1.575) for the 2nd, 3rd, and 4th quartile, respectively, compared with the 1st quartile (*p* for trend < 0.001, Table 2). Also, in a multivariate-adjusted model for age, sex, smoking, alcohol consumption, regular physical activity, low socioeconomic status, BMI, hypertension, and total cholesterol level, there was a significant and progressive increase in the risk of stroke, MI, and both with increasing TyG index quartile.

**Table 2:** Risk of stroke, myocardial infarction, and both according to TyG index quartile

TyG index	Events (*n*)	Duration (person-years)	Incidence rate (per 1000 person-years)	Model 1, hazard ratio (95% CI)	Model 2, hazard ratio (95% CI)	Model 3, hazard ratio (95% CI)
Myocardial infarction and stroke						
Q1	26,773	11,447,594.33	2.33874	1 (ref.)	1 (ref.)	1(ref.)
Q2	33,335	11,430,071.25	2.91643	1.247 (1.227, 1.267)	1.18 (1.161, 1.199)	1.085 (1.068, 1.103)
Q3	39,119	11,416,557.3	3.42651	1.466 (1.443, 1.489)	1.338 (1.317, 1.359)	1.157 (1.138, 1.176)
Q4	47,517	11,384,076.25	4.17399	1.786 (1.759, 1.813)	1.608 (1.584, 1.633)	1.282 (1.262, 1.304)
*p* for trend				< .0001	< .0001	< .0001
Myocardial infarction						
Q1	11,223	11,503,460.68	0.97562	1 (ref.)	1 (ref.)	1 (ref.)
Q2	14,124	11,500,096.33	1.22816	1.257 (1.226, 1.289)	1.204 (1.174, 1.234)	1.092 (1.065, 1.12)
Q3	16,618	11,499,511.73	1.4451	1.48 (1.444, 1.516)	1.379 (1.346, 1.412)	1.165 (1.137, 1.194)
Q4	20,612	11,483,933.86	1.79486	1.837 (1.795, 1.88)	1.697 (1.658, 1.737)	1.312 (1.28, 1.345)
*p* for trend				< .0001	< .0001	< .0001
Stroke						
Q1	16,378	11,483,024.16	1.42628	1 (ref.)	1 (ref.)	1 (ref.)
Q2	20,315	11,474,799.11	1.7704	1.242 (1.217, 1.268)	1.164 (1.14, 1.188)	1.080 (1.058, 1.103)
Q3	23,806	11,469,602.92	2.07557	1.457 (1.428, 1.486)	1.306 (1.28, 1.333)	1.148 (1.125, 1.172)
Q4	28,621	11,450,595.06	2.49952	1.754 (1.72, 1.788)	1.545 (1.515, 1.575)	1.260 (1.234, 1.287)
*p* for trend				< .0001	< .0001	< .0001

Model 1: crude

Model 2: adjusted for age and sex

Model 3: adjusted for age, sex, smoking, alcohol consumption, regular physical activity, low socioeconomic status, body mass index, hypertension, total cholesterol level, hypertension medications, warfarin, and aspirin

### Sensitivity analysis: effects of clinical variables and number of risk factors on associations of TyG index with stroke, myocardial infarction, and both

**Fig. 2:**
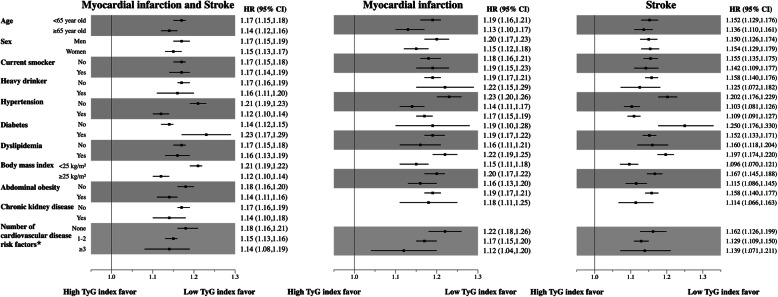
Risk of myocardial infarction, stroke, and both according to a prespecified subgroup comparing the highest TyG index quartile with all others after adjusting for age, sex, smoking, alcohol consumption, regular physical activity, low economic status, and total cholesterol level. *Current smoking, hypertension, diabetes, dyslipidemia, chronic kidney disease. HR, hazard ratio; CI, confidence interval; TyG, triglyceride glucose

Associations of TyG index with stroke, myocardial infarction, and both were generally consistent across subgroups according to clinical variables, including known individual cardiovascular risk factors, after multivariate adjustment (Fig. 2). We also classified the population according to cardiovascular risk based on the number of cardiovascular risk factors present (current smoking, hypertension, diabetes, dyslipidemia, and chronic kidney disease) as “none,” “1–2,” and “3 or more.” Regardless of the number of cardiovascular risk factors present, associations of TyG index with stroke, myocardial infarction, and both were generally consistent. The risks of incident stroke (*p* for interaction < 0.001), myocardial infarction (*p* for interaction < 0.002), and both (*p* for interaction < 0.001) for the 4th quartile of TyG index in the “None” subgroup were significantly higher than those of the “1–2” and “3 or more” subgroups. We also conducted a sensitivity analysis without excluding participants who were taking hypoglycemic drugs and lipid-lowering drugs. The results of sensitivity analysis were consistent with those of the primary analysis (Additional file 1: Table S1).

## Discussion

This present study investigated the association between TyG index and ASCVD using a longitudinal national representative cohort dataset. High TyG index was found to be associated with a significantly increased risk of future ASCVD events, and this finding persisted even after adjusting for possible confounders such as cardiovascular risk factors. Notably, these findings were seen in all subgroup analyses, further emphasizing the consistency of this association. Although the results of an observational study should be interpreted with caution, this large nationwide observational study with 5,593,134 participants showed that high TyG index could be a significant predictor of future cardiovascular events.

Some previous studies also have shown an association between TyG index and ASCVD. A 10-year follow-up study of the Vascular Metabolic CUN cohort with 5014 patients showed that higher TyG index was significantly associated with an increased risk of new ASCVD, and TyG index could provide additive value to the Framingham risk score for predicting ASCVD [18]. Another recent study with 12,326 participants from the Korea Initiatives on Coronary Artery Calcification registry showed that TyG index is an independent predictor of coronary artery calcification progression [24]. Consistent with previous studies, we confirmed that higher TyG index was significantly associated with increased risk of future myocardial infarction and stroke. Previous studies showed that TyG index was a useful marker in patients at 10% to 20% 10-year risk [18] or without heavy baseline coronary artery calcification [24]. We found that TyG index was consistently useful for predicting myocardial infarction, stroke, and both regardless of the presence, absence, or number of cardiovascular risk factors (Fig. 2). Although these associations were more prominent in populations with a low number of cardiovascular risk factors, our study demonstrated that TyG index could predict the development of ASCVD events across all populations.

Patients with insulin resistance are unable to mount a normal coordinated glucose-lowering response involving suppression of endogenous glucose production, suppression of lipolysis, cellular uptake of available plasma glucose, and net glycogen synthesis at a normal plasma insulin level [25, 26]. At the onset of insulin resistance, increased lipolysis of stored triglycerides in adipose tissue produces more fatty acids before elevating plasma glucose level. Three decades ago, Reaven et al. reported that insulin resistance stimulates de novo lipogenesis, leading to increased triglycerides even in normo-triglyceridemic non-obese participants and correlates with plasma triglyceride level [27]. Based on the above rationale, the TyG index, a product of fasting blood glucose and triglyceride levels, has been suggested as a simple alternative surrogate marker of insulin resistance. Guerrero-Romero et al. reported that TyG index, regardless of glucose tolerance and obesity, inversely correlates with insulin resistance determined using the euglycemic-hyperinsulinemic clamp test as the gold standard method [22]. In another study, TyG index showed better performance in predicting insulin resistance using the hyperglycemic clamp test compared with the homeostasis model assessment insulin resistance (HOMA-IR) index [28]. Multiple studies have shown that insulin resistance is a strong predictor of ASCVD [29–33]. In a recent meta-analysis of 65 studies involving 516,325 participants, the relative risk of cardiovascular disease was higher for an increase of one standard deviation in HOMA-IR compared to an increase of one standard deviation in fasting glucose or fasting insulin concentration [34]. We also observed that higher insulin resistance determined by the TyG index was significantly associated with higher risk of future ASCVD events.

The strengths of our study are that we used a large-scale nationwide database representing the entire Korean population. Second, we conducted fully adjusted analyses with all available confounding factors and sensitivity analysis for the absence, presence, and number of cardiovascular risk factors. Third, the findings of the present study suggest that TyG index could be a suitable marker for screening high ASCVD risk populations in developing countries, because it is a low-cost index. However, this study also has some limitations. First, the retrospective observational study design had inherent limitations. Although the analyses were adjusted for most available demographic and clinical variables, some unidentified parameters could have affected the results. Second, we defined MI and stroke with claims data; this may not be a totally accurate method for determining the number of cases. To overcome this problem, we defined outcomes with the operational definition by combining diagnosis and prescription records. Last, we did not directly measure insulin resistance. While there may be some discrepancies between TyG index and insulin resistance, performing the hyperinsulinemic-euglycemic clamp test on 5,593,134 participants was not feasible. However, a previous study established correlations between TyG index and insulin resistance and cardiovascular risk factors via the hyperinsulinemic-euglycemic clamp test, the gold standard method for measurement of insulin resistance [35].

## Conclusion

In conclusion, we demonstrated that a higher TyG index is associated with a higher risk of ASCVD using a longitudinal national representative cohort dataset. This, however, does not indicate a causal relationship because of the inherent limitations of the observational study design. Our results suggest that TyG index, as a surrogate marker of insulin resistance, may be an independent predictor of ASCVD development.

## Supplementary Information

**Additional file 1: Table S1.** Risk of stroke, myocardial infarction, and both according to TyG index quartile without excluding participants taking hypoglycemic or lipid-lowering drugs. MODEL 1: Crude. MODEL 2: Adjusted for age and sex. MODEL 3: Adjusted for age, sex, smoking, alcohol consumption, regular physical activity, low socioeconomic status, body mass index, hypertension, and total cholesterol level. MODEL 4: Adjusted for age, sex, smoking, alcohol consumption, regular physical activity, low socioeconomic status, body mass index, hypertension, total cholesterol level, hypertension medication, warfarin, aspirin, hypoglycemic drugs, and statin.

## Data Availability

The data that supports the findings of this study are available from the Korean National Health Insurance Service (KNHIS), but restrictions apply to the availability of the data, which were used with permission for the current study and therefore not publicly available. Data are however available from the corresponding author upon reasonable request and with permission from KNHIS.

## Abbreviations

ASCVD: Atherosclerotic cardiovascular disease
BP: Blood pressure
CKD: Chronic kidney disease
CI: Confidential interval
FBG: Fasting blood glucose
HR: Hazard ratio
HDL: High-density lipoprotein
HOMA-IR: Homeostasis model assessment insulin resistance
NHID: National Health Information Database
NHIS: National Health Insurance Service
LDL: Low-density lipoprotein
MI: Myocardial infarction
TG: Triglyceride
TyG: Triglyceride glucose
