# Supplementary material for: The triglyceride glucose index is a simple and low-cost marker associated with atherosclerotic cardiovascular disease: a population-based study
Source: BMC Med. 2020 Nov 25;18:361. doi: 10.1186/s12916-020-01824-2 (PMC7687762; doi:10.1186/s12916-020-01824-2)
Supplement: Supplementary file 1 — Additional file 1: Table S1. Risk of stroke, myocardial infarction, and both according to TyG index quartile without excluding participants taking hypoglycemic or lipid-lowering drugs. MODEL 1: Crude. MODEL 2: Adjusted for age and sex. MODEL 3: Adjusted for age, sex, smoking, alcohol consumption, regular physical activity, low socioeconomic status, body mass index, hypertension, and total cholesterol level. MODEL 4: Adjusted for age, sex, smoking, alcohol consumption, regular physical activity, low socioeconomic status, body mass index, hypertension, total cholesterol level, hypertension medication, warfarin, aspirin, hypoglycemic drugs, and statin. [file 12916_2020_1824_MOESM1_ESM.docx]

Table S1. Risk of stroke, myocardial infarction, and both according to TyG index quartile without excluding participants taking hypoglycemic or lipid-lowering drugs

| TyG index | Number of participants | Events (n) | Duration (person-years) | Incidence rate (per 1000 person-years) | MODEL 1 | MODEL 2 | MODEL 3 | MODEL4 |
| --- | --- | --- | --- | --- | --- | --- | --- | --- |
| Myocardial infarction and stroke | | | | | Hazard ratio (95% CI) | Hazard ratio (95% CI) | Hazard ratio (95% CI) | Hazard ratio (95% CI) |
| Q1 | 1695567 | 100631 | 13857808.42 | 7.2617 | 1(Ref.) | 1(Ref.) | 1(Ref.) | 1(Ref.) |
| Q2 | 1696267 | 113466 | 13829412.87 | 8.2047 | 1.13(1.12,1.139) | 1.047(1.038,1.056) | 1.046(1.037,1.055) | 1.033(1.024,1.042) |
| Q3 | 1696344 | 124954 | 13798390.22 | 9.0557 | 1.247(1.237,1.257) | 1.106(1.097,1.115) | 1.105(1.096,1.115) | 1.073(1.064,1.083) |
| Q4 | 1695974 | 148577 | 13714488.56 | 10.8336 | 1.492(1.481,1.504) | 1.314(1.304,1.325) | 1.3(1.288,1.311) | 1.202(1.192,1.213) |
| p for trend |  |  |  |  | <.0001 | <.0001 | <.0001 | <.0001 |
| Myocardial infarction | | | | |  |  |  |  |
| Q1 | 1695567 | 15330 | 13935591.85 | 1.10006 | 1(Ref.) | 1(Ref.) | 1(Ref.) | 1(Ref.) |
| Q2 | 1696267 | 19936 | 13931931.69 | 1.43096 | 1.3(1.273,1.327) | 1.222(1.196,1.248) | 1.115(1.092,1.139) | 1.098(1.075,1.122) |
| Q3 | 1696344 | 24400 | 13922494.27 | 1.75256 | 1.591(1.56,1.624) | 1.439(1.41,1.469) | 1.229(1.203,1.255) | 1.186(1.162,1.212) |
| Q4 | 1695974 | 31607 | 13878552.33 | 2.2774 | 2.067(2.028,2.108) | 1.835(1.799,1.871) | 1.443(1.414,1.474) | 1.316(1.288,1.344) |
| p for trend |  |  |  |  | <.0001 | <.0001 | <.0001 | <.0001 |
| Stroke | | | | |  |  |  |  |
| Q1 | 1695567 | 22322 | 13907323.71 | 1.60505 | 1(Ref.) | 1(Ref.) | 1(Ref.) | 1(Ref.) |
| Q2 | 1696267 | 28987 | 13894301.43 | 2.08625 | 1.299(1.277,1.322) | 1.191(1.17,1.212) | 1.11(1.09,1.13) | 1.093(1.074,1.113) |
| Q3 | 1696344 | 34790 | 13878856.9 | 2.50669 | 1.561(1.535,1.587) | 1.35(1.327,1.373) | 1.193(1.172,1.214) | 1.152(1.132,1.172) |
| Q4 | 1695974 | 45343 | 13820028.64 | 3.28096 | 2.042(2.01,2.075) | 1.719(1.691,1.748) | 1.417(1.392,1.442) | 1.29(1.268,1.314) |
| p for trend |  |  |  |  | <.0001 | <.0001 | <.0001 | <.0001 |

MODEL 1: Crude

MODEL 2: Adjusted for age and sex

MODEL 3: Adjusted for age, sex, smoking, alcohol consumption, regular physical activity, low socioeconomic status, body mass index, hypertension, and total cholesterol level

MODEL 4: Adjusted for age, sex, smoking, alcohol consumption, regular physical activity, low socioeconomic status, body mass index, hypertension, total cholesterol level, hypertension medication, warfarin, aspirin, hypoglycemic drugs, and statin
